# Development of Novel antimiRzymes for Targeted Inhibition of miR-21 Expression in Solid Cancer Cells

**DOI:** 10.3390/molecules24132489

**Published:** 2019-07-07

**Authors:** Leon M. Larcher, Tao Wang, Rakesh N. Veedu

**Affiliations:** 1 Centre for Molecular Medicine and Innovative Therapeutics, Murdoch University, Perth, WA 6150, Australia; 2 Perron Institute for Neurological and Translational Science, Perth, WA 6009, Australia

**Keywords:** oligonucleotides, DNAzyme, antimiRzyme, miR-21 targeting

## Abstract

MicroRNAs (miRNAs) are short non-coding RNAs that are involved in the regulation of gene expression. Previous reports showed an over-expression of miRNA-21 (miR-21) in various cancer cells, and its up-regulation is closely related to cancer initiation, proliferation and metastasis. In this work, we envisioned the development of novel antimiRzymes (anti-miRNA-DNAzyme) that are capable of selectively targeting and cleaving miR-21 and inhibit its expression in cancer cells using the DNAzyme technique. For this purpose, we have designed different antimiRzyme candidates by systematically targeting different regions of miR-21. Our results demonstrated that RNV541, a potential arm-loop-arm type antimiRzyme, was very efficient (90%) to suppress miR-21 expression in U87MG malignant glioblastoma cell line at 200 nM concentration. In addition, RNV541 also inhibited miR-21 expression (50%) in MDA-MB-231 breast cancer cell line. For targeted delivery, we conjugated RNV541 with a transferrin receptor (TfR) targeting aptamer for TfR-mediated cancer cell delivery. As expected, the developed chimeric structure efficiently delivered the antimiRzyme RNV541 into TfR positive glioblastoma cells. TfR aptamer-RNV541 chimeric construct showed 52% inhibition of miR-21 expression in U87MG glioblastoma cells at 2000 nM concentration, without using any transfection reagents, making it a highly desirable strategy to tackle miR-21 over-expressed malignant cancers. Although these are in vitro based observations, based on our results, we firmly believe that our findings could be beneficial towards the development of targeted cancer therapeutics where conventional therapies face several challenges.

## 1. Introduction

MicroRNAs (miRNAs) are 18–25 nucleotides (nt) long non-protein coding RNAs that are involved in the regulation of gene expression. Growing evidence in recent years has demonstrated that tissue-specific expression patterns of miRNAs directly correlate with altered expression profiles of up to 90% of human gene transcripts [1], and are associated with the development of more than 160 different types of diseases [2] including cancers. Indeed, dysregulation of miRNA has been reported in the key steps of the cancer development such as initiation, proliferation, immune invasion and metastasis. Recent in vivo studies demonstrated that restoration of aberrantly expressed miRNAs could alleviate and even reverse the malignant cancer phenotypes [3]. Over the past decade, a number of miRNAs have been reported to be dysregulated in human cancers. MiRNA 21 (miR-21) is one of the most investigated candidates, and which is over-expressed in a variety of cancer cells including glioma [4], breast cancer [5], hepatocellular carcinoma [6], pancreatic cancer, colorectal cancer [7], lung cancer, oral cancer [8], and acute myeloid leukemia [9], as well as various cancer stem cells [10]. Importantly, miR-21 has been confirmed to be critical in the maintenance of the malignant phenotype of cancer and inversely correlated with patient survival and tumor grading [11,12,13]. Consequently, miR-21 has gained significant interest in the development of cancer therapeutics. Various miRNA targeting strategies have been reported in recent years towards developing novel therapeutic molecules. Application of antisense oligonucleotide sequences commonly known as antimiRs (or antagomiRs) or miRNA sponges are some of the common direct methods for miRNA inhibition [14]. However, recent reports also demonstrated indirect inhibition of miRNA biogenesis using CRISPER (Clustered Regularly Interspaced Short Palindromic Repeats) system [15] and also by using small molecules [16]. Although these approaches are effective, there are some disadvantages, such as efficacy and tissue specific delivery, that need to be considered for their clinical translations [16].

DNA enzymes (commonly known as DNAzymes) are a unique class of single-stranded catalytic oligonucleotide that can specifically target messenger RNAs through Watson–Crick base pairing interactions and inhibit gene expression [17]. The structure of the DNAzyme includes two binding arms for target specific mRNA binding, and a catalytic loop connecting the binding arms designed to cleave the phosphodiester bond in the target RNA mainly at the purine–pyrimidine or, less frequently, at the purine–purine junction in the presence of divalent metal ions (e.g., Mg^2+^, Ca^2+^, Pb^2+^) [18]. The unique structural properties of DNAzymes ensure the high-specific gene regulation compared with other gene silencing methods such as siRNA and antisense oligonucleotides [18]. Although DNAzymes are majorly used to target messenger RNAs, this type of molecule can also be used to target and inhibit miRNAs. Towards this, Belter et al. reported the inhibition of miRNAs in glioma cells [19]. Compared with other strategies currently used for miRNA inhibition, DNAzyme technology has certain advantages, such as low production cost for DNA oligonucleotide synthesis, and, importantly, the target RNA cleavage process is repetitive over several cycles of annealing and catalytic cleavage, which in theory will require a low concentration of DNAzyme for sustainable therapeutic efficacy [19]. In this study, we envisioned the design and development of novel catalytic oligonucleotides targeting miR-21 termed antimiRzymes to inhibit their expression in solid cancers, and then developed a transferrin receptor (TFR) targeting aptamer conjugated antimiRzyme chimera for targeted inhibition of miR-21 without using any lipid-based transfection reagents.

## 2. Results

### 2.1. Rational Design and In Vitro Evaluation of antimiRzymes Targeting miR-21

**Figure 1:**
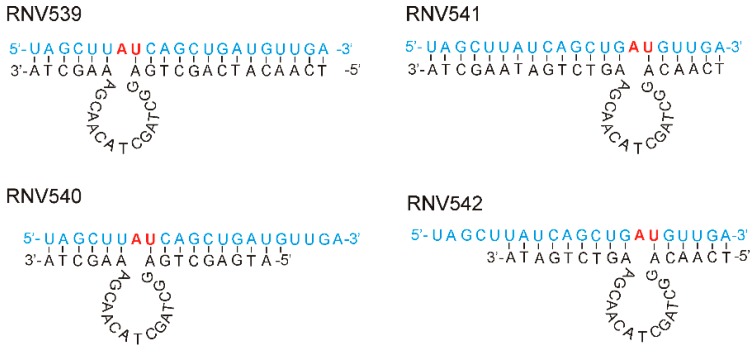
Design of novel “10–23” motif antimiRzymes RNV539, RNV540, RNV541 and RNV542 targeting miR-21. The designed antimiRzymes had a 15 nt catalytic loop and two substrate binding arms of varying sizes. In this design, the antimiRzymes (black) and its miR-21substrate (blue) is hybridized via Watson–Crick base pairing and cleavage occurs at the A:U dinucleotide position (red).

DNAzymes with “8–17” and “10–23” catalytic motif structures developed by in vitro selection methodologies are the two widely studied and well-characterized RNA-cleaving DNA molecules [20]. The 10–23 DNAzyme structure cleaves the RNA at any purine–pyrimidine (RY) junction under physiological conditions, and showed better tolerance to different target sequences [20]. In our studies, we used this DNAzyme pattern to design novel antimiRzymes specific to miR-21. Santoro et al. suggested that the highest cleavage efficiency was observed with ‘A:U’ and ‘G:U’ combinations [21]. In line with this, we selected two different ‘A:U’ sites of miR-21-5p sequence for designing antimiRzymes. As shown in Figure 1, four different DNAzyme sequences, namely, RNV539, RNV540, RNV541 and RNV542, with varying binding arm lengths at both 5′- and 3′-ends were designed, and each contained a conserved 15-mer “10–23” catalytic motif. The 5′ and 3′ binding arm sequences were designed to form Watson–Crick base pairs specific to the mature miR-21target. 

**Figure 2:**
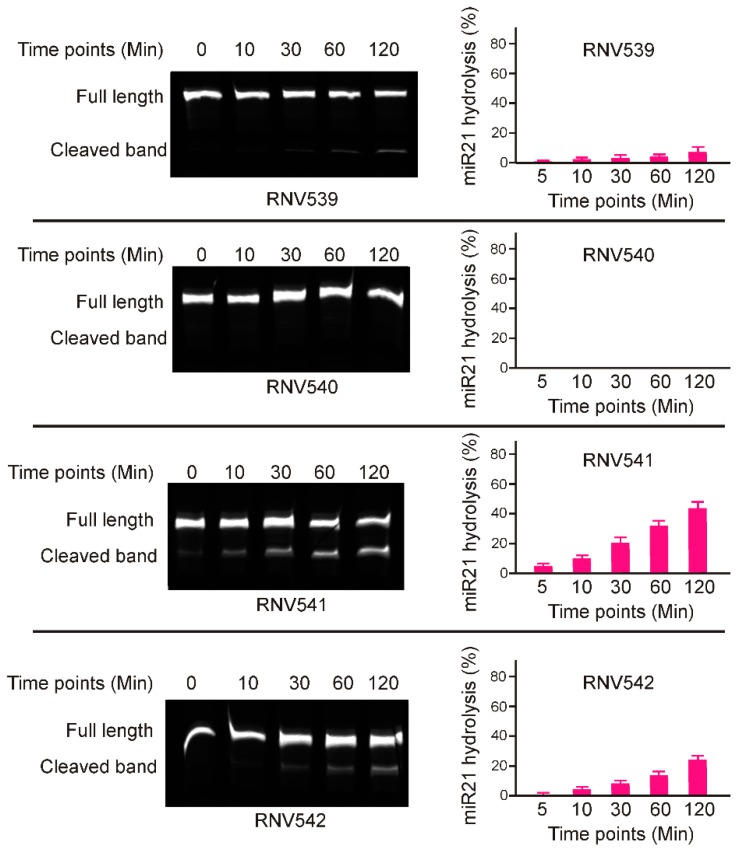
In vitro miR-21 RNA template cleavage assay using the designed synthetic antimiRzymes. Synthetic miR-21 RNA template was incubated with 100 nM antimiRzymes at 37 °C. The reactions were terminated by adding formamide solution at the indicated time points. The products were then separated on a 15% denaturing polyacrylamide gel and visualized using Fusion FX Bilber Lourmat imager. The densitometry analysis was performed using the ImageJ program.

Although the designed antimiRzymes share an identical catalytic domain, the miR-21 cleaving efficacy of individual antimiRzyme sequences could be affected by various other factors such as targeting affinity, capabilities to adopt the arm-loop-arm structure, and catalytic properties in the presence of divalent metal ions [20], and this has to be experimentally tested. To determine the catalytic activity of the antimiRzymes, initially we performed in vitro cleavage experiments by incubating the antimiRzymes RNV539-RNV542 with a 5′-fluorescent dye (FAM) labelled synthetic miR-21 RNA template in presence of 10 mM Mg^2+^ divalent cations at different time points over two hours. The results demonstrated that both RNV541 and 542 displayed notable miR-21 cleavage in a time-dependent manner (Figure 2), whereas RNV539 and RNV540 were not effective in cleaving miR-21 RNA template as it showed low or no catalytic efficacy. In particular, RNV541 showed 40% cleavage efficiency after 120 min incubation which was almost twice the cleavage rate achieved with RNV542. In line with these initial results of miR-21 RNA template cleavage, we have used the antimiRzyme RNV541 for further analyses in miR-21 overexpressed cancer cells, including glioblastoma U87MG and triple negative breast cancer MDA-MB-231 cells. 

### 2.2. Evaluation of miR-21 Cleavage Activity of RNV541 antimiRzymes in Cancer Cell Lines

**Figure 3:**
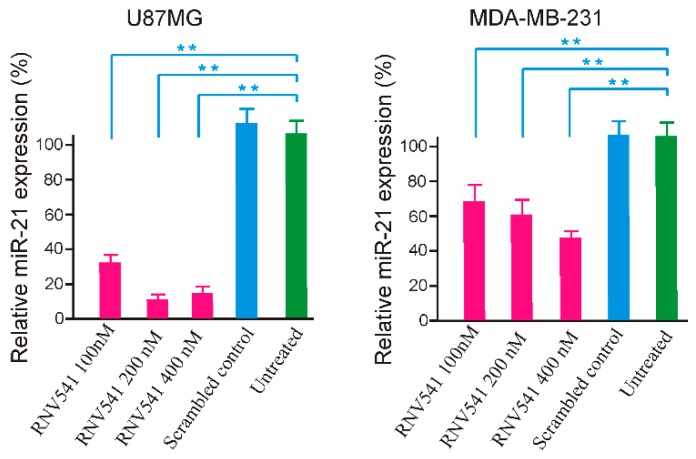
miR-21 cleavage efficacy of RNV541 antimiRzymes on U87MG glioblastoma and MDA-MB-231 breast cancer cells. Cells were transfected with RNV541 at the indicated concentration for 24 h using Lipofectamine 3000. The total miRNAs were then isolated, and miR-21 expression was quantified by performing real-time PCR assay. ** *p* < 0.01.

Following the initial in vitro screening using synthetic miR-21 template, we investigated the potential of RNV541 to target and cleave miR-21 in cancer cells by transfecting RNV541 in glioblastoma (U87MG) and breast cancer (MDA-MB-231) cell lines at different concentrations of 100 nM, 200 nM and 400 nM. After 24 h of transfection, the cells were harvested, and total RNA was isolated. The expression of miR-21 was measured by RT-qPCR. Notably, RNV541 demonstrated remarkable reduction in mi-21 expression in U87MG glioblastoma cells. Specifically, we observed 68% and 90% reduction in miR-21 expression at 100 nM and 200 nM concentrations, respectively (Figure 3). The cleavage efficacy seemed to have plateaued at 200 nM as no further improvement in cleavage was observed at 400 nM. In addition, we also observed high inhibition of miR-21 expression in MDA-MB-231 breast cancer cells after the treatment with RNV541. In the case of MDA-MB-231 cells, a similar dose dependent miR-21 reduction was observed, however, the efficacy of miR-21 inhibition was not as high as observed in U87MG cells, but moderately high with rates of 32%, 40% and 54% for 100 nM, 200 nM and 400 nM concentrations, respectively. 

### 2.3. Evaluation of Transferrin Receptor Aptamer-RNV541 Chimera for Cancer Cell Targeted Inhibition of miR-21

Targeted delivery is one of the major challenges in oligonucleotide drug development. For high therapeutic efficacy in vivo, the antimiRzymes need to specifically target and penetrate cancer cells in high concentration for a sufficient period of time, which remains a significant hurdle for oligonucleotide-based cancer treatment. Generally, the negative charge of nucleic acid structures makes them difficult to penetrate or diffuse across the lipid cell membranes [20] in order to recognize their RNA targets in the cytoplasm. Towards this goal, nucleic acid aptamer technology provides a great opportunity to circumvent the targeted delivery issue of antimiRzymes. Aptamers are short single-stranded nucleic acid sequences that can recognize and bind to target molecules with high affinity and specificity in a way similar to antibodies [22,23,24]. Various aptamer-mediated drug delivery systems have been explored in recent years for either small molecule chemotherapeutics [25] or therapeutic nucleic acids (i.e., siRNA, antisense oligonucleotide, antimiR) [26,27]. In this work, we aimed at delivering miR-21 into cancer cells by employing a previously developed transferrin receptor (TfR) aptamer [28]. 

**Figure 4:**
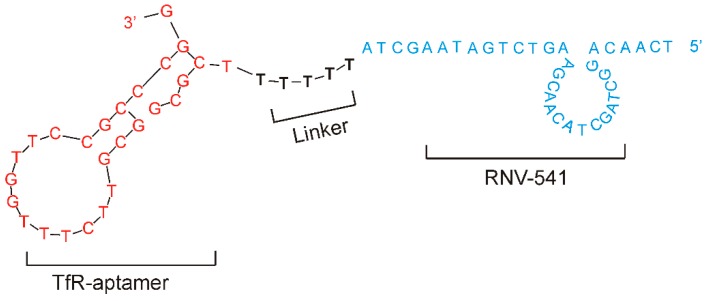
Schematic illustration of TfR aptamer-RNV541 antimiRzyme chimera. The chimeric structure consisted of TfR aptamer (red) and RNV-541 DNAzyme (blue) sections, which were jointed via a d(TTTTT) linker.

Although TfR is generally expressed at low levels in different tissues under physiological conditions [29], TfR over-expression has been observed in different cancer cells [30]. Importantly, via clathrin-mediated endocytosis, TfR binding aptamers can mediate the traversal of TfR conjugated molecules into cancer cells [31], making them a promising target for precision cancer therapy. As shown in Figure 4, we conjugated the TfR aptamer to RNV541 antimiRzyme via a five consecutive deoxythymidine nucleotides (-TTTTT-) bridge to construct TfR aptamer-RNV541 chimera. As reported previously, the TTTTT linker could help retaining the structural conformation of both the TfR aptamer and the antimiRzyme RNV541 [32]. While the purpose of the aptamer part is to specifically target TfR and internalize into cancer cells efficiently, the RNV541 antimiRzyme component will recognize and inhibit the expression of endogenous miR-21 target in the cytoplasm. 

### 2.4. TfR Aptamer-RNV541 Chimera Effectively Suppressed miR-21 Expression in Glioblastoma Cells

**Figure 5:**
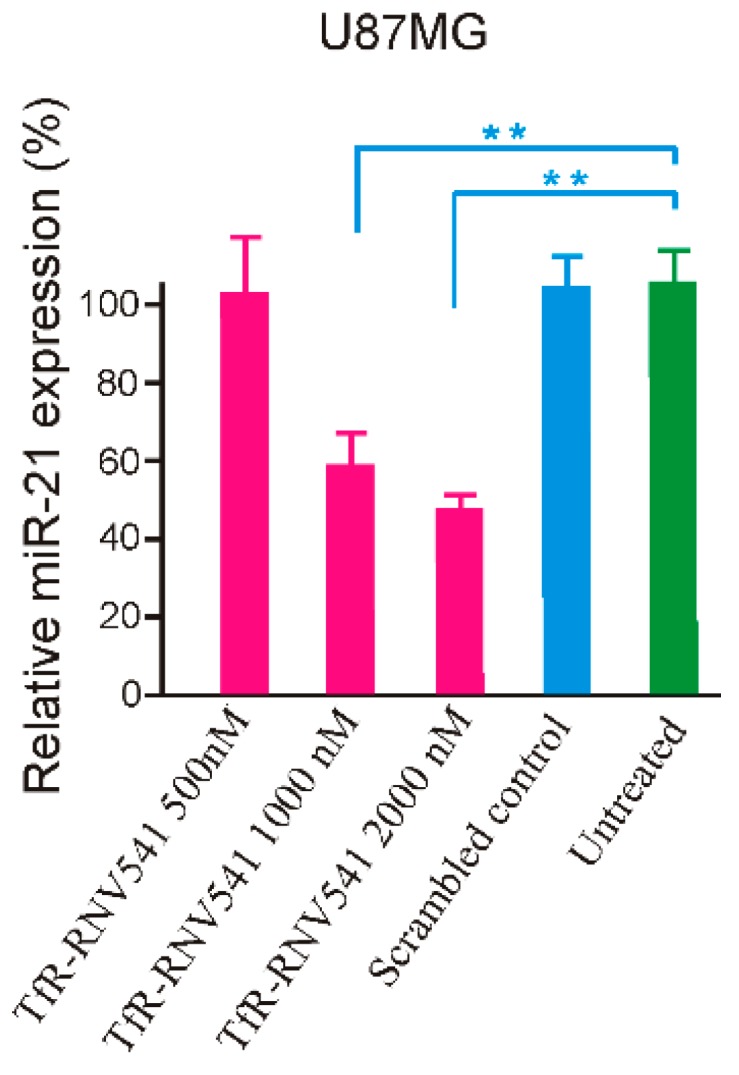
Evaluation of miR-21 inhibition using TfR aptamer-RNV541 antimiRzyme chimera in U87MGglioblastoma cells. Cells were treated with TfR aptamer-RNV541 chimera at the indicated concentrations for 24 h in the absence of transfection reagents. The total miRNAs were then isolated and the quantification of miR-21 expression was conducted via RT-qPCR. ** *p* < 0.01.

The developed TfR aptamer-RNV541 chimera was then synthesized and tested in the TfR positive U87MG glioblastoma cells without using any transfection reagents. Our results demonstrated that the TfR aptamer-RNV541 chimera showed a clear dose-dependent inhibition of miR-21 expression in U87MG cells (Figure 5). Higher inhibition of miR-21 expression was observed at both 1000 nM (41% inhibition) and 2000 nM (52% inhibition) treatment groups. However, it should be mentioned that the unconjugated RNV541 did not show any change in reducing miR-21 expression (data not shown). These results provide proof-of-principle evidence regarding the rationality of utilizing an aptamer conjugated antimiRzyme approach for targeted inhibition of miR-21 in malignant glioblastoma cells. 

## 3. Discussion

Despite the progress in cancer management over the past century, conventional treatments including surgery, radiation, and chemotherapy still face a number of challenges [33,34]. As a relatively new field, tumor suppressor miRNAs, such as miR-21, miR-155, miR-10, have demonstrated great potential in future cancer treatment [35]. Compared with traditional cancer treatment, miRNA-mediated anti-cancer therapy enables regulation of entire signaling networks within the cells, making them an interesting option for developing cancer therapeutics [36]. The reported “OncomiR Addiction” depicts the restoration of certain dysregulated miRNAs capable of abrogating and even reversing the malignant cancer phenotypes [37]. This is especially true for miR-21, one of the prominent and most investigated “oncomiRs” [37]. A well-designed recent in vivo study demonstrated complete regression of malignant lymphoid-like phenotype tumors after the inactivation of miR-21 [37]. Further investigations also disclosed that miR-21 is expressed in virtually all types of cancer cells and the miR-21 upregulation is closely correlated with all key steps of cancer development, making it a highly desirable target to tackle malignant cancers [38]. Previous efforts on catalytic DNA molecules (DNAzymes) mainly focused on the regulation of messenger RNAs, and recently the potential of DNAzyme was also applied for miRNA regulation [39]. As DNAzyme-mediated miRNA silencing possesses certain advantages over traditional miRNA regulation strategies, we investigated the potential of catalytic DNA molecules, termed antimiRzymes, to target and cleave miR-21, using the established “10–23” arm-loop-arm design containing a defined 15-mer catalytic core. Since there is no GU position at the purine–pyrimidine junction at the catalytic cleavage site on the mature miR-21 sequence, two of the available AU points on miR-21 sequence were used to design four different antimiRzyme candidates named RNV539–RNV542 (Figure 1). By performing an in vitro cleavage assay, we found that only two candidates, RNV541 and RNV542, targeting the A:U junction at the 3′ end, achieved notable miR-21 cleavage efficacy, of which RNV541 was found to more effective with 40% cleavage efficacy (Figure 2). As suggested by a previous study, we speculate that the observed variable efficiencies of different antimiRzyme sequences might be due to the formation of improper secondary structures ultimately affecting the potential of the conserved catalytic core region [19]. 

Inspired by the in vitro cleavage assay, we extended the evaluation of RNV541 in malignant glioblastoma (U87MG) and breast cancer cells (MDA-MB-231), and the experiment demonstrated that RNV541 sequence was remarkably effective in inhibiting the expression of miR-21 in U87MG cells (90%), and also yielded 54% inhibition in MDA-MB-231 cells. As reported previously, a single miRNA has the ability to target and regulate hundreds of genes [3], and therefore, even small perturbations in the miRNA network may have an impact on cell phenotype. Taking this into account, antimiRzyme sequence RNV541, with a 90% miR-21 inhibition rate in cancer cells, may hold great potential to be developed as a novel anti-cancer therapeutic. However, similar to other nucleic acid-based medicines, delivery remains a key issue for the systemic administration of antimiRzymes. Although antibodies have been employed for targeted delivery, their clinical translation is impeded to some extent by severe side effects and immunogenicity. To overcome this, we used nucleic acid aptamer technology towards the delivery of antimiRzyme RNV541. To successfully deliver antimiRzyme into cancer cells, an aptamer not only needs to recognize an appropriate cancer marker, but also requires efficient cell internalization after target binding [40,41]. The previously developed TfR aptamer represents an ideal tool for this purpose [42]. TfR is a type of membrane glycoprotein responsible for iron homeostasis [31]. While TfR is ubiquitously expressed at low level in most normal human tissues, highly elevated expression of TfR is typically associated with rapidly proliferating cells, mainly in cancers [29,30]. This is not surprising given that malignant cells have an intrinsically high demand for iron as a cofactor for DNA synthesis and cell cycle progression. The significance of TfR in targeted cancer therapy is supported by several factors: (i) overexpression by cancer cells, (ii) cell surface accessibility promoted by clathrin/dynamin dependent endocytosis [31], and (iii) an essential role in cell growth and proliferation. Over the past decades, TfR-based drug delivery systems have been explored for glioblastoma, breast cancer and Alzheimer’s disease [42,43]. 

Similar to previously reported aptamer-therapeutic nucleic acid chimeras [44], a bifunctional aptamer-antimiRzyme chimera was designed in this study by utilizing a previously reported TfR aptamer [42] and the newly developed antimiRzyme RNV541. A 5-mer consecutive 2′-deoxy thymidine nucleotide (-TTTTT-) was used to link the TfR aptamer and the RNV541 antimiRzyme in order to construct the chimera, which might reduce any possible interaction between the aptamer and antimiRzymes, and can also maximize the possibility of retaining the functions of both TfR aptamer and RNV541 (Figure 4). After testing with TfR overexpressed glioblastoma U87MG cells in the absence of transfection reagents, over 50% miR-21 suppression was recorded, which clearly demonstrates the efficacy of aptamer-RNV541 chimera. Unlike other therapeutic nucleic acid-based approaches, such as siRNA, which only interrupts and regulates a particular type of gene, antimiRzyme RNV541-based inhibition of miR-21 can affect post transcriptional regulation of the miR-21 network by controlling the expression of hundreds of genes implicated in cancer pathology. This suggests that even small-scale suppression of the miR-21 expression could result in a significant anti-cancer effect, and the 50% miR-21 suppression rate observed for aptamer-RNV541 chimera without using any transfection reagent is therefore very promising towards targeted cancer treatment. 

## 4. Materials and Methods

### 4.1. Oligonucleotide Sequences 

All sequences were synthesized by Integrated DNA Technologies (IDT, Singapore, Singapore). The sequence information of RNV539–542 is shown in Figure 1, and the aptamer-antimiRzyme chimera is shown in Figure 4 (before treatment, this chimera structure was denatured at 95 °C for 5 min and then snap cooled on ice for 10 min). Both antimiRzymes and aptamer-RNV541 antimiRzyme chimera sequences were synthesized in house on GE AKTA oligopilot plus 10 oligonucleotide synthesizer (GE Healthcare Life Sciences, Sydney, Australia) using standard phosphoramidite chemistry in 1 µmol scale. 

### 4.2. Cell Culture and In Vitro Cleavage Assay 

U87MG (human malignant glioblastoma) cells were purchased from Cell Bank Australia (Sydney, Australia). MDA-MB-231 cells (ATCC, Manassas, VA, USA) were kindly provided by A/Prof. Stacey Edwards at the Queensland Berghofer Institute for Medical Research. All cells were cultured at 37 °C in Dulbecco’s Modified Eagle Media (ThermoFisher, 12491-015, Melbourne, Australia) supplemented with 10% FBS (Sigma, F8192, Sydney, Australia) and supplying 5% CO_2_/air. 

A quantity of 20 µM mature miRNA was incubated with 100 nM antimiRzymes in the presence of 10 mM Mg^2+^ divalent cations for 30 min, 60 min, 120 min, and 180 min at 37 °C. The reactions were terminated by adding 10 µL of formamide solutions. The products were then separated on a 15% denaturing polyacrylamide gel and visualized using a Fusion FX Bilber Lourmat imager. 

### 4.3. Transfection

Lipofectamine 3000 (L3000001, Invitrogen, Melbourne, Australia) was used according to the manufacturer’s instructions. Briefly, cells were added on T25 cm^2^ flasks at a density of 2 × 10^5^ cells/mL in the indicated growth medium and propagated to 80% confluence at the time of transfection. The antimiRzymes and control sequences were mixed with Lipofectamine 3000 with 1 µg nucleic acid per 2 µL of Lipofectamine 3000 [TR/DNA ratio (*w*/*w*) = 2:1] and 1.5 µL of P3000 in the Opti-MEM Reduced Serum Medium. The cells were then incubated at 37 °C for 24 h. All transfection assays were carried out independently and with an untreated group and scrambled sequence group as controls. 

### 4.4. Taqman qPCR to Measure the Expression of miR-21

Twenty-four hours after transfection of miR-21 targeting antimiRzymes, the total RNA of different treatments was harvested, and cDNA was prepared by TaqMan™ MicroRNA Reverse Transcription Kit (4427975, ThermoFisher, Melbourne, Australia) according to the supplier’s specifications. The gene-specific miR-21 primer is designed to match with the hsa-miR-21-5p sequence of UAGCUUAUCAGACUGAUGUUGA. Real-time PCR was preformed using TaqMan Universal Master Mix (4440040, ThermoFisher, Melbourne, Australia). on a C1000™ Thermal cycler, CFX96™ real-time system (BioRad, Sydney, Australia) and programmed initially at 95 °C for 10 min, 95 °C for 15 s, then 60 °C for 1 min, and repeated for a total of 40 cycles.

### 4.5. Statistical Analysis

All statistical analyses were performed using GraphPad Prism 3.03. An unpaired t test was used for comparisons between two experimental groups. Unless otherwise indicated, all results were averaged from biological triplicates and the values are reported as means ± SEM. A *p*-value of less than 0.01 was considered statistically significant.

## 5. Conclusions

In summary, we investigated the potential of novel antimiRzymes for targeted inhibition of miR-21, one of the most promising oncomiRs implicated in cancer pathology. A novel arm-loop-arm type antimiRzyme RNV541 showed efficient inhibition of miR-21 in vitro and also in U87MG glioblastoma cells and MDA-MB-231 triple negative breast cancer cells. Based on the high efficiency in glioblastoma cells, targeted delivery of RNV541 was investigated using the previously reported transferrin receptor targeting aptamer. The developed TfR aptamer-RNV541 chimera effectively delivered the RNV541 and showed efficient inhibition of miR-21 expression. Although we need to demonstrate the potential of RNV541 in vivo, based on our very promising results, we firmly believe that this work could serve as a proof-of-concept for the potential development of aptamer guided antimiRzymes towards clinical development.
